# Confirming the Importance of the R-Spine: New Insights into Protein Kinase Regulation

**DOI:** 10.1371/journal.pbio.1001681

**Published:** 2013-10-15

**Authors:** Richard Robinson

**Affiliations:** Freelance Science Writer, Sherborn, Massachusetts, United States of America

[Fig pbio-1001681-g001]Phosphorylation is a ubiquitous means of changing a protein's behavior. Adding a phosphate group to a serine, threonine, or tyrosine amino acid alters the shape and charge of the protein, a change that may activate an enzyme, promote membrane translocation, or trigger binding to DNA. Protein kinases—enzymes that add phosphates to other proteins—are so important to eukaryotic cell function that they account for about 2% of all human genes, and act on almost a third of all human proteins. Aberrant protein kinase activity drives a myriad of diseases, and, not surprisingly, these protein kinases are central targets for a wide variety of medications, including drugs for cancer, heart disease, and diabetes.

**Figure pbio-1001681-g001:**
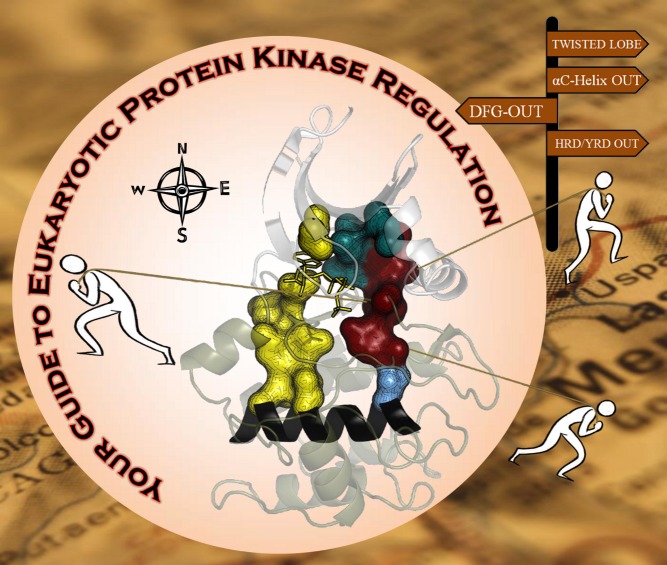
Eukaryotic protein kinase regulation. Structural motifs in eukaryotic protein kinases adopt specific conformations indicative of whether the kinase is in an active or inactive state; knowledge of these conformations may aid in identifying specific kinase inhibitors.

The requirements of rational drug design, as well as pure human curiosity, have led researchers to try to understand the structural features of protein kinases that contribute to their regulation. In this issue of *PLOS Biology*, Hiruy Meharena, Alexandr Kornev, and colleagues demonstrate the central importance of one structural element common to all protein kinases, and identify key amino acids that contribute to the stability of the catalytic core.

The structural core of eukaryotic protein kinases is highly conserved, and consists of two lobes, called the N-terminal and C-terminal lobes (N-lobe and C-lobe). The cleft between them forms the active site. The core is further characterized by a functional element called the catalytic spine (C-spine) formed from several structurally distinct parts of the two lobes. The unusual characteristic of the C-spine is that the adenine ring of ATP (the source of phosphate) is a part of the hydrophobic stack formed by the amino acid side chains.

In work predating the discovery of the C-spine, the authors suggested that several other noncontiguous sections of the two lobes created a separate motif, parallel to the C-spine, that was also essential for catalysis, which they termed the regulatory spine (R-spine). Unlike the C-spine, the R-spine is highly regulated, which typically involves phosphorylation of a portion of the R-spine called the activation loop. Here, they rigorously tested the importance of the R-spine by systematically mutating multiple key residues within it to determine the effect on catalytic activity, using as a model cyclic AMP-dependent protein kinase.

The researchers began by aligning sequences of more than 13,000 eukaryotic protein kinases, to determine which amino acid residues of the R-spine were conserved. They focused on four in particular, termed RS1, RS2, RS3, and RS4. RS1 was an aromatic amino acid, either histidine or tyrosine in virtually all protein kinases, while RS2 was typically also an aromatic residue, usually phenylalanine. RS3 and RS4 were almost always hydrophobic. When the normal amino acids at RS1 or RS2 were replaced with hydrophilic amino acids, catalytic activity was abolished. Switching RS3 or RS4 to hydrophilic amino acids had much less of an effect. They saw the same pattern of high sensitivity of RS1 and RS2, low sensitivity of RS3 and RS4, when testing the importance of the specific hydrophobic side chain atoms. Replacing the normal, more elaborate side chains with smaller hydrophobic groups drastically reduced catalytic activity in the first pair, but not the second pair.

The reason for that difference in response, they suspected, lay in the local environment. RS1 and RS2 are part of the C-lobe, while RS3 and RS4 are part of the N-lobe. Of 14 amino acids nearby RS3 and RS4, only three are highly conserved, all of them hydrophobic. Because they seemed to be supporting the N-lobe region of the R-spine, the authors dubbed these three the Shell (Sh). They found, for instance, that mutation of RS3 to alanine had little effect unless Sh2 was also changed, from methionine to glycine. Replacement of the bulky methionine side chain with the small methyl side chain of alanine almost completely eliminated catalytic activity.

Finally, the authors examined 172 publicly available structures of protein kinases, and found four different conformations in which the R-spine was disassembled, corresponding to inactive states of the respective enzymes, strengthening the evidence that the R-spine is central for catalytic activity, and broadening the understanding of protein kinase activity regulation.

These results will likely have large and almost immediate practical implications. There are currently two dozen protein kinase inhibitors approved for clinical use, and many more in various stages of development. The confirmation that the R-spine is critical for catalytic activity, and the identification of the most sensitive residues within it, is likely to expand the array of targets for the development of new inhibitors. In addition, mutations in these same kinases are responsible for multiple human diseases, and better understanding of the pathogenic consequences of those mutations may lead to a more rational approach to therapies designed to restore the function of the mutant proteins.

**Meharena HS, Chang P, Keshwani MM, Oruganty K, Nene AK, et al. (2013) Deciphering the Structural Basis of Eukaryotic Protein Kinase Regulation. doi:10.1371/journal.pbio.1001680**

